# [2,2′-Dihy­droxy-*N* ^2^,*N* ^2′^-(3-hy­droxy­imino­pentane-2,4-di­yl)dibenzo­hydra­zid­ato]copper(II)

**DOI:** 10.1107/S1600536810040754

**Published:** 2010-10-20

**Authors:** Zi-Jing Xiao

**Affiliations:** a College of Materials Science & Engineering, Huaqiao University, Quanzhou 362021, People’s Republic of China

## Abstract

The Cu^II^ atom in the title complex, [Cu(C_19_H_17_N_5_O_5_)], is coordinated by two N atoms and two O atoms of one 2,2′-dihy­droxy-*N* ^2^,*N* ^2^’-(3-hy­droxy­imino­pentane-2,4-di­yl)dibenzo­hydrazidate ligand, exhibiting a distorted square-planar geometry. The dihedral angle between the two benzene rings in the oxime hydrazone is 7.62 (15)°. The molecular configuration is stabilized by intramolecular O—H⋯N hydrogen bonds. Pairs of centrosymmetrically related molecules are linked into dimers by two inter­molecular C—H⋯O hydrogen bonds. Each dimer is further connected to four neighboring dimers *via* four O—H⋯O hydrogen bonds, forming an extended two-dimensional structure. The oxime O atom is disordered over two orientations in a 2:1 ratio.

## Related literature

For the structural versatility and biological activity of oxime hydrazone compounds and their metal complexes, see: Marmion *et al.* (2004 ▶); Song *et al.* (2000 ▶); Xiao *et al.* (2004 ▶). For similar copper(II) complexes with Schiff bases, see: Suleiman Gwaram *et al.* (2010 ▶); Qin *et al.* (2010 ▶).
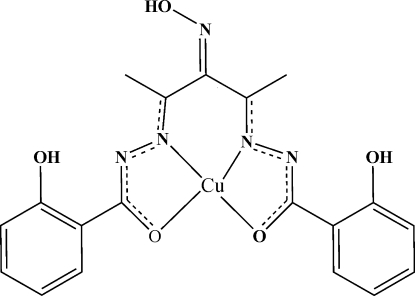


## Experimental

### 

#### Crystal data

[Cu(C_19_H_17_N_5_O_5_)]
*M* *_r_* = 458.92
Monoclinic, 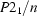

*a* = 8.7672 (5) Å
*b* = 19.0262 (11) Å
*c* = 11.6118 (6) Å
β = 91.437 (2)°
*V* = 1936.31 (19) Å^3^
*Z* = 4
Mo *K*α radiation
μ = 1.17 mm^−1^
*T* = 293 K
0.52 × 0.22 × 0.15 mm

#### Data collection

Rigaku Weissenberg IP diffractometer
Absorption correction: multi-scan (*TEXRAY*; Molecular Structure Corporation, 1999 ▶) *T*_min_ = 0.715, *T*_max_ = 0.833
17167 measured reflections
4436 independent reflections
3065 reflections with *I* > 2σ(*I*)
*R*_int_ = 0.037

#### Refinement

*R*[*F* ^2^ > 2σ(*F* ^2^)] = 0.034
*wR*(*F* ^2^) = 0.079
*S* = 0.91
4436 reflections
282 parameters
H-atom parameters constrained
Δρ_max_ = 0.39 e Å^−3^
Δρ_min_ = −0.40 e Å^−3^

### 

Data collection: *TEXRAY* (Molecular Structure Corporation, 1999 ▶); cell refinement: *TEXRAY*; data reduction: *TEXSAN* (Mol­ecular Structure Corporation, 1999 ▶); program(s) used to solve structure: *SHELXS97* (Sheldrick, 2008 ▶); program(s) used to refine structure: *SHELXL97* (Sheldrick, 2008 ▶); molecular graphics: *ORTEX* (McArdle, 1995 ▶); software used to prepare material for publication: *SHELXL97*.

## Supplementary Material

Crystal structure: contains datablocks I, global. DOI: 10.1107/S1600536810040754/om2355sup1.cif

Structure factors: contains datablocks I. DOI: 10.1107/S1600536810040754/om2355Isup2.hkl

Additional supplementary materials:  crystallographic information; 3D view; checkCIF report

## Figures and Tables

**Table 1:** Hydrogen-bond geometry (Å, °)

*D*—H⋯*A*	*D*—H	H⋯*A*	*D*⋯*A*	*D*—H⋯*A*
O1—H1*A*⋯N1	0.82	1.86	2.586 (2)	147
O5—H5*B*⋯N5	0.82	1.79	2.519 (2)	148
C19—H19*A*⋯O2^i^	0.96	2.58	3.525 (3)	170
O3*A*⋯O5^ii^			2.643 (3)	

Symmetry codes: (i) 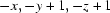; (ii) 
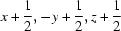
.

**Fig. 1.**
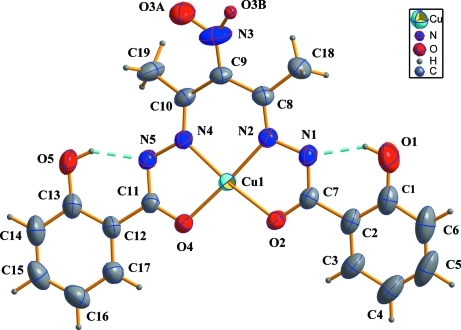
The molecular structure of the title complex, with the atom-numbering scheme. Displacement ellipsoids are drawn at the 50% probability level. Dashed lines indicate intramolecular hydrogen bonding.

**Fig. 2.**
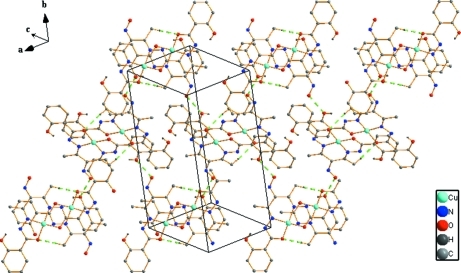
The extended two-dimensional layer of the title complex, dotted green lines represent intermolecular hydrogen bonding. Only H atoms participating in hydrogen bonds are shown.

## supplementary crystallographic information

### Comment

Recently, much attention has been focused on oxime compounds and their complexes, due to their biological activities, chemical and industrial versatility, and excellent chelating capability (Marmion, *et al.*, 2004; Song *et al.*, 2000; Xiao, *et al.*, 2004). Oxime hydrazone and hydroxamic acids are the two kinds of oxime compounds. Herein, we present the synthesis and structure of copper complex with 2,2'-dihydroxy-*N*^2^,*N*^2^'-(3-hydroxyiminopentane- 2,4-diyl)dibenzohydrazide.

As shown in Figure 1, the copper atom in the title complex has a square-planar coordination geometry, the four donors are two hydrazine nitrogen atoms and two carboxyl oxygen atoms in the oxime hydrazone ligand. The bond lengths of Cu-N and Cu-O in the square planar complex are similar to those in this type of copper complex (Suleiman Gwaram *et al.*, 2010; Qin *et al.*, 2010). The whole oxime hydrazone ligand is not planar; the dihedral angle between the two benzene rings in the oxime hydrazone ligand is 7.62 (15)°.

There are intramolecular O-H ···N hydrogen bonds, and intermolecular oxime-phenol O-H···O hydrogen bonds and intermolecular C-H···O hydrogen bonds in the title complex. The interatomic distance of O3A – O5^ii^ (symmetry code ii, 1/2+x, 1/2-y, 1/2+z) is 2.643 (3)Å. It means that there may exist O-H (oxime group) ···O (phenol) hydrogen bonds, although the hydrogen atom in N-O-H has not been found. These intermolecular O-H···O and C-H···O hydrogen bonds result in an extended two-dimensional layer structure (see Figure 2).

### Experimental

Solid CuBr_2_ (0.0477g, 0.2 mmol) was dissolved in 12 mL of methanol to get solution A. 2, 3, 4-pentanetrione-3-oxime (0.0258g, 0.2 mmol) and salicyloyl hydrazine (0.0304g, 0.2 mmol) were dissolved in 12 mL of methanol to get solution B. To the solution B was added the solution A slowly. The reaction mixture was stirred and refluxed for 2 hours to give a green solution. The deep blue crystals of the title complex were formed upon slow evaporation at about 297 K after 20 d.

### Refinement

On the residial density there is one peak, 2.24 e Å3, corresponding to a second position for the O3 atom. The treatment of the disorder shows that the O3 atom in the N3-O3 oxime group is disordered over two postions as O3A and O3B in ratio 2/1. The oxime group-bound H atom was not found in the difference Fourier map. All other H atoms were placed in calculated positions and refined using a riding model [C-H = 0.93 Å and Uiso(H) = 1.2Ueq(C) for aromatic H atoms, C-H = 0.96 Å and Uiso(H) = 1.5Ueq(C) for methyl H atoms, and O-H = 0.82 Å and Uiso(H) = 1.2Ueq(O)].

### Crystal data

**Table:**

[Cu(C_19_H_17_N_5_O_5_)]	*F*(000) = 940
*M**_r_* = 458.92	*D*_x_ = 1.574 Mg m^−^^3^
Monoclinic, *P*2_1_/*n*	Mo *K*α radiation, λ = 0.71073 Å
Hall symbol: -P 2yn	Cell parameters from 4577 reflections
*a* = 8.7672 (5) Å	θ = 2.1–27.5°
*b* = 19.0262 (11) Å	µ = 1.17 mm^−^^1^
*c* = 11.6118 (6) Å	*T* = 293 K
β = 91.437 (2)°	Prism, blue
*V* = 1936.31 (19) Å^3^	0.52 × 0.22 × 0.15 mm
*Z* = 4	

### Data collection

**Table:**

Rigaku Weissenberg IP diffractometer	4436 independent reflections
Radiation source: fine-focus sealed tube	3065 reflections with *I* > 2σ(*I*)
graphite	*R*_int_ = 0.037
ω scans	θ_max_ = 27.5°, θ_min_ = 2.1°
Absorption correction: multi-scan (*TEXRAY*; Molecular Structure Corporation, 1999)	*h* = 0→11
*T*_min_ = 0.715, *T*_max_ = 0.833	*k* = −24→24
17167 measured reflections	*l* = −15→15

### Refinement

**Table:**

Refinement on *F*^2^	Primary atom site location: structure-invariant direct methods
Least-squares matrix: full	Secondary atom site location: difference Fourier map
*R*[*F*^2^ > 2σ(*F*^2^)] = 0.034	Hydrogen site location: inferred from neighbouring sites
*wR*(*F*^2^) = 0.079	H-atom parameters constrained
*S* = 0.91	*w* = 1/[σ^2^(*F*_o_^2^) + (0.040*P*)^2^] where *P* = (*F*_o_^2^ + 2*F*_c_^2^)/3
4436 reflections	(Δ/σ)_max_ = 0.002
282 parameters	Δρ_max_ = 0.39 e Å^−^^3^
0 restraints	Δρ_min_ = −0.40 e Å^−^^3^

### Special details

**Table:**

Geometry. All esds (except the esd in the dihedral angle between two l.s. planes) are estimated using the full covariance matrix. The cell esds are taken into account individually in the estimation of esds in distances, angles and torsion angles; correlations between esds in cell parameters are only used when they are defined by crystal symmetry. An approximate (isotropic) treatment of cell esds is used for estimating esds involving l.s. planes.
Refinement. Refinement of F^2^ against ALL reflections. The weighted R-factor wR and goodness of fit S are based on F^2^, conventional R-factors R are based on F, with F set to zero for negative F^2^. The threshold expression of F^2^ > 2sigma(F^2^) is used only for calculating R-factors(gt) etc. and is not relevant to the choice of reflections for refinement. R-factors based on F^2^ are statistically about twice as large as those based on F, and R- factors based on ALL data will be even larger.

### Fractional atomic coordinates and isotropic or equivalent isotropic displacement parameters (Å^2^)

**Table:**

	*x*	*y*	*z*	*U*_iso_*/*U*_eq_	Occ. (<1)
Cu1	0.24832 (3)	0.508624 (12)	0.47601 (2)	0.03770 (9)	
N1	0.46486 (19)	0.53298 (9)	0.64875 (15)	0.0428 (4)	
N2	0.36561 (18)	0.48024 (9)	0.61023 (14)	0.0380 (4)	
N3	0.2905 (3)	0.30306 (12)	0.6725 (2)	0.0735 (7)	
N4	0.13118 (18)	0.42316 (8)	0.47217 (14)	0.0364 (4)	
N5	0.01862 (18)	0.42494 (9)	0.38607 (15)	0.0401 (4)	
O1	0.6627 (2)	0.59621 (11)	0.78120 (17)	0.0805 (6)	
H1A	0.6074	0.5640	0.7587	0.097*	
O2	0.34881 (15)	0.59685 (7)	0.50147 (13)	0.0414 (3)	
O3A	0.2209 (4)	0.24814 (15)	0.6492 (3)	0.0749 (8)	0.67
O3B	0.3899 (6)	0.2757 (2)	0.7190 (5)	0.0635 (15)	0.33
O4	0.12933 (16)	0.53067 (7)	0.33856 (12)	0.0414 (3)	
O5	−0.16592 (19)	0.36688 (8)	0.24617 (14)	0.0604 (5)	
H5B	−0.1070	0.3703	0.3019	0.072*	
C1	0.6346 (3)	0.65344 (14)	0.7173 (2)	0.0581 (7)	
C2	0.5294 (2)	0.65449 (12)	0.6241 (2)	0.0461 (5)	
C3	0.5081 (3)	0.71686 (12)	0.5633 (2)	0.0594 (7)	
H3A	0.4376	0.7182	0.5022	0.071*	
C4	0.5894 (3)	0.77687 (15)	0.5916 (3)	0.0784 (9)	
H4A	0.5743	0.8181	0.5498	0.094*	
C5	0.6921 (4)	0.77491 (18)	0.6816 (3)	0.0911 (11)	
H5A	0.7467	0.8153	0.7011	0.109*	
C6	0.7166 (3)	0.71460 (19)	0.7439 (3)	0.0849 (10)	
H6A	0.7882	0.7144	0.8044	0.102*	
C7	0.4416 (2)	0.59175 (11)	0.58840 (18)	0.0386 (5)	
C8	0.3669 (2)	0.42120 (11)	0.66539 (18)	0.0409 (5)	
C9	0.2605 (2)	0.36376 (11)	0.62829 (19)	0.0405 (5)	
C10	0.1317 (2)	0.37065 (10)	0.54304 (19)	0.0394 (5)	
C11	0.0336 (2)	0.48128 (10)	0.31893 (17)	0.0355 (4)	
C12	−0.0688 (2)	0.48412 (11)	0.21619 (17)	0.0402 (5)	
C13	−0.1615 (3)	0.42678 (13)	0.1830 (2)	0.0485 (5)	
C14	−0.2529 (3)	0.43151 (16)	0.0835 (2)	0.0647 (7)	
H14A	−0.3146	0.3939	0.0613	0.078*	
C15	−0.2519 (3)	0.49149 (17)	0.0182 (2)	0.0718 (8)	
H15A	−0.3126	0.4939	−0.0485	0.086*	
C16	−0.1628 (3)	0.54807 (16)	0.0496 (2)	0.0679 (7)	
H16A	−0.1637	0.5886	0.0047	0.082*	
C17	−0.0720 (3)	0.54442 (13)	0.14814 (19)	0.0510 (6)	
H17A	−0.0120	0.5828	0.1695	0.061*	
C18	0.4736 (3)	0.41084 (14)	0.7671 (2)	0.0635 (7)	
H18A	0.4647	0.4498	0.8190	0.095*	
H18B	0.5765	0.4079	0.7412	0.095*	
H18C	0.4478	0.3682	0.8062	0.095*	
C19	0.0055 (3)	0.31770 (13)	0.5377 (3)	0.0628 (7)	
H19A	−0.0886	0.3407	0.5167	0.094*	
H19B	−0.0038	0.2958	0.6117	0.094*	
H19C	0.0278	0.2826	0.4812	0.094*	

### Atomic displacement parameters (Å^2^)

**Table:**

	*U*^11^	*U*^22^	*U*^33^	*U*^12^	*U*^13^	*U*^23^
Cu1	0.03850 (14)	0.03025 (14)	0.04393 (15)	−0.00304 (11)	−0.00734 (9)	0.00278 (11)
N1	0.0401 (9)	0.0385 (10)	0.0495 (11)	−0.0008 (8)	−0.0091 (8)	−0.0045 (8)
N2	0.0340 (8)	0.0354 (10)	0.0443 (10)	0.0032 (7)	−0.0031 (7)	−0.0007 (8)
N3	0.0906 (18)	0.0425 (13)	0.0885 (18)	0.0125 (12)	0.0220 (14)	0.0210 (12)
N4	0.0365 (9)	0.0305 (9)	0.0418 (10)	0.0005 (7)	−0.0045 (7)	−0.0002 (7)
N5	0.0402 (9)	0.0339 (9)	0.0459 (10)	−0.0028 (7)	−0.0059 (8)	−0.0011 (8)
O1	0.0776 (13)	0.0845 (15)	0.0776 (14)	−0.0080 (11)	−0.0329 (11)	−0.0108 (11)
O2	0.0395 (8)	0.0317 (8)	0.0527 (9)	−0.0020 (6)	−0.0074 (7)	0.0017 (6)
O3A	0.093 (2)	0.0467 (17)	0.084 (2)	0.0043 (15)	−0.0252 (17)	0.0092 (14)
O3B	0.072 (3)	0.037 (3)	0.079 (4)	0.012 (2)	−0.045 (3)	0.014 (2)
O4	0.0468 (8)	0.0340 (8)	0.0430 (8)	−0.0071 (7)	−0.0071 (6)	0.0029 (6)
O5	0.0683 (11)	0.0463 (10)	0.0655 (11)	−0.0133 (8)	−0.0179 (9)	−0.0114 (8)
C1	0.0483 (14)	0.0602 (17)	0.0656 (17)	−0.0051 (12)	−0.0011 (12)	−0.0208 (13)
C2	0.0377 (11)	0.0423 (13)	0.0586 (14)	−0.0036 (10)	0.0049 (10)	−0.0157 (11)
C3	0.0614 (16)	0.0400 (14)	0.0772 (18)	−0.0083 (12)	0.0089 (13)	−0.0106 (12)
C4	0.083 (2)	0.0457 (16)	0.107 (3)	−0.0180 (15)	0.0168 (19)	−0.0154 (16)
C5	0.083 (2)	0.068 (2)	0.123 (3)	−0.0347 (18)	0.013 (2)	−0.040 (2)
C6	0.0618 (19)	0.094 (3)	0.098 (2)	−0.0187 (18)	−0.0068 (17)	−0.044 (2)
C7	0.0328 (10)	0.0360 (11)	0.0471 (13)	0.0016 (8)	0.0023 (9)	−0.0067 (9)
C8	0.0383 (11)	0.0382 (11)	0.0461 (13)	0.0094 (9)	−0.0020 (9)	0.0026 (9)
C9	0.0414 (11)	0.0332 (11)	0.0468 (12)	0.0057 (9)	0.0012 (9)	0.0072 (9)
C10	0.0377 (11)	0.0288 (10)	0.0517 (13)	0.0036 (8)	0.0040 (9)	0.0010 (9)
C11	0.0362 (10)	0.0325 (10)	0.0377 (11)	0.0030 (8)	−0.0009 (8)	−0.0051 (8)
C12	0.0394 (11)	0.0439 (12)	0.0370 (11)	0.0012 (9)	−0.0011 (8)	−0.0068 (9)
C13	0.0475 (13)	0.0499 (14)	0.0479 (13)	−0.0009 (10)	−0.0040 (10)	−0.0118 (11)
C14	0.0592 (15)	0.076 (2)	0.0583 (16)	−0.0082 (14)	−0.0143 (12)	−0.0202 (15)
C15	0.0670 (17)	0.098 (2)	0.0495 (14)	−0.0008 (17)	−0.0213 (12)	−0.0039 (16)
C16	0.0711 (17)	0.082 (2)	0.0497 (15)	0.0027 (16)	−0.0104 (13)	0.0140 (14)
C17	0.0506 (13)	0.0555 (15)	0.0466 (13)	−0.0031 (11)	−0.0061 (10)	0.0035 (11)
C18	0.0716 (17)	0.0571 (16)	0.0605 (16)	0.0002 (13)	−0.0220 (13)	0.0119 (13)
C19	0.0526 (14)	0.0424 (14)	0.093 (2)	−0.0080 (11)	−0.0113 (14)	0.0233 (13)

### Geometric parameters (Å, °)

**Table:**

Cu1—O2	1.9153 (13)	C4—C5	1.362 (4)
Cu1—N2	1.9230 (16)	C4—H4A	0.9300
Cu1—N4	1.9231 (16)	C5—C6	1.371 (5)
Cu1—O4	1.9308 (14)	C5—H5A	0.9300
N1—C7	1.333 (3)	C6—H6A	0.9300
N1—N2	1.395 (2)	C8—C9	1.493 (3)
N2—C8	1.293 (3)	C8—C18	1.501 (3)
N3—O3B	1.140 (4)	C9—C10	1.489 (3)
N3—O3A	1.236 (3)	C10—C19	1.497 (3)
N3—C9	1.288 (3)	C11—C12	1.476 (3)
N4—C10	1.294 (3)	C12—C17	1.393 (3)
N4—N5	1.388 (2)	C12—C13	1.408 (3)
N5—C11	1.334 (3)	C13—C14	1.392 (3)
O1—C1	1.337 (3)	C14—C15	1.370 (4)
O1—H1A	0.8200	C14—H14A	0.9300
O2—C7	1.284 (2)	C15—C16	1.374 (4)
O3A—O3B	1.752 (5)	C15—H15A	0.9300
O4—C11	1.277 (2)	C16—C17	1.379 (3)
O5—C13	1.356 (3)	C16—H16A	0.9300
O5—H5B	0.8200	C17—H17A	0.9300
C1—C6	1.398 (4)	C18—H18A	0.9600
C1—C2	1.404 (3)	C18—H18B	0.9600
C2—C3	1.391 (3)	C18—H18C	0.9600
C2—C7	1.474 (3)	C19—H19A	0.9600
C3—C4	1.381 (3)	C19—H19B	0.9600
C3—H3A	0.9300	C19—H19C	0.9600
			
O2—Cu1—N2	83.48 (6)	N2—C8—C9	119.70 (18)
O2—Cu1—N4	171.09 (7)	N2—C8—C18	120.1 (2)
N2—Cu1—N4	93.19 (7)	C9—C8—C18	120.22 (19)
O2—Cu1—O4	100.02 (6)	N3—C9—C10	119.2 (2)
N2—Cu1—O4	176.23 (6)	N3—C9—C8	114.9 (2)
N4—Cu1—O4	83.54 (6)	C10—C9—C8	125.81 (18)
C7—N1—N2	110.44 (16)	N4—C10—C9	118.78 (18)
C8—N2—N1	117.96 (17)	N4—C10—C19	120.15 (19)
C8—N2—Cu1	130.03 (15)	C9—C10—C19	121.07 (19)
N1—N2—Cu1	111.99 (13)	O4—C11—N5	124.12 (18)
O3B—N3—O3A	94.9 (3)	O4—C11—C12	120.09 (18)
O3B—N3—C9	138.1 (4)	N5—C11—C12	115.79 (18)
O3A—N3—C9	125.1 (3)	C17—C12—C13	118.7 (2)
C10—N4—N5	117.91 (17)	C17—C12—C11	119.46 (19)
C10—N4—Cu1	130.13 (14)	C13—C12—C11	121.9 (2)
N5—N4—Cu1	111.46 (12)	O5—C13—C14	118.6 (2)
C11—N5—N4	111.23 (16)	O5—C13—C12	121.8 (2)
C1—O1—H1A	109.5	C14—C13—C12	119.5 (2)
C7—O2—Cu1	109.58 (13)	C15—C14—C13	120.1 (2)
C11—O4—Cu1	109.08 (13)	C15—C14—H14A	119.9
C13—O5—H5B	109.5	C13—C14—H14A	119.9
O1—C1—C6	117.9 (3)	C14—C15—C16	121.1 (2)
O1—C1—C2	123.2 (2)	C14—C15—H15A	119.4
C6—C1—C2	118.9 (3)	C16—C15—H15A	119.4
C3—C2—C1	118.7 (2)	C15—C16—C17	119.6 (3)
C3—C2—C7	119.0 (2)	C15—C16—H16A	120.2
C1—C2—C7	122.2 (2)	C17—C16—H16A	120.2
C4—C3—C2	121.5 (3)	C16—C17—C12	121.0 (2)
C4—C3—H3A	119.2	C16—C17—H17A	119.5
C2—C3—H3A	119.2	C12—C17—H17A	119.5
C5—C4—C3	119.1 (3)	C8—C18—H18A	109.5
C5—C4—H4A	120.4	C8—C18—H18B	109.5
C3—C4—H4A	120.4	H18A—C18—H18B	109.5
C4—C5—C6	121.3 (3)	C8—C18—H18C	109.5
C4—C5—H5A	119.3	H18A—C18—H18C	109.5
C6—C5—H5A	119.3	H18B—C18—H18C	109.5
C5—C6—C1	120.5 (3)	C10—C19—H19A	109.5
C5—C6—H6A	119.8	C10—C19—H19B	109.5
C1—C6—H6A	119.8	H19A—C19—H19B	109.5
O2—C7—N1	124.22 (19)	C10—C19—H19C	109.5
O2—C7—C2	118.43 (19)	H19A—C19—H19C	109.5
N1—C7—C2	117.36 (19)	H19B—C19—H19C	109.5
			
C7—N1—N2—C8	−175.43 (18)	Cu1—N2—C8—C9	−2.6 (3)
C7—N1—N2—Cu1	5.9 (2)	N1—N2—C8—C18	−0.3 (3)
O2—Cu1—N2—C8	177.17 (19)	Cu1—N2—C8—C18	178.08 (16)
N4—Cu1—N2—C8	5.46 (19)	O3B—N3—C9—C10	160.8 (5)
O2—Cu1—N2—N1	−4.33 (12)	O3A—N3—C9—C10	0.8 (4)
N4—Cu1—N2—N1	−176.04 (13)	O3B—N3—C9—C8	−16.5 (6)
N2—Cu1—N4—C10	4.20 (19)	O3A—N3—C9—C8	−176.5 (3)
O4—Cu1—N4—C10	−177.69 (19)	N2—C8—C9—N3	166.7 (2)
N2—Cu1—N4—N5	175.78 (13)	C18—C8—C9—N3	−14.0 (3)
O4—Cu1—N4—N5	−6.10 (12)	N2—C8—C9—C10	−10.4 (3)
C10—N4—N5—C11	−179.12 (18)	C18—C8—C9—C10	168.9 (2)
Cu1—N4—N5—C11	8.1 (2)	N5—N4—C10—C9	173.72 (17)
N2—Cu1—O2—C7	1.81 (13)	Cu1—N4—C10—C9	−15.1 (3)
O4—Cu1—O2—C7	−176.79 (13)	N5—N4—C10—C19	−5.6 (3)
C9—N3—O3A—O3B	166.8 (5)	Cu1—N4—C10—C19	165.55 (17)
C9—N3—O3B—O3A	−163.7 (6)	N3—C9—C10—N4	−157.7 (2)
O2—Cu1—O4—C11	−169.01 (12)	C8—C9—C10—N4	19.3 (3)
N4—Cu1—O4—C11	2.74 (13)	N3—C9—C10—C19	21.6 (3)
O1—C1—C2—C3	179.8 (2)	C8—C9—C10—C19	−161.4 (2)
C6—C1—C2—C3	−1.4 (4)	Cu1—O4—C11—N5	1.5 (2)
O1—C1—C2—C7	−0.6 (4)	Cu1—O4—C11—C12	−178.11 (14)
C6—C1—C2—C7	178.3 (2)	N4—N5—C11—O4	−6.6 (3)
C1—C2—C3—C4	0.9 (4)	N4—N5—C11—C12	172.98 (16)
C7—C2—C3—C4	−178.7 (2)	O4—C11—C12—C17	−7.1 (3)
C2—C3—C4—C5	−0.4 (4)	N5—C11—C12—C17	173.30 (19)
C3—C4—C5—C6	0.3 (5)	O4—C11—C12—C13	171.42 (19)
C4—C5—C6—C1	−0.8 (5)	N5—C11—C12—C13	−8.2 (3)
O1—C1—C6—C5	−179.8 (3)	C17—C12—C13—O5	−178.9 (2)
C2—C1—C6—C5	1.3 (4)	C11—C12—C13—O5	2.6 (3)
Cu1—O2—C7—N1	1.3 (2)	C17—C12—C13—C14	0.3 (3)
Cu1—O2—C7—C2	−179.02 (15)	C11—C12—C13—C14	−178.2 (2)
N2—N1—C7—O2	−4.9 (3)	O5—C13—C14—C15	179.5 (2)
N2—N1—C7—C2	175.42 (17)	C12—C13—C14—C15	0.3 (4)
C3—C2—C7—O2	0.4 (3)	C13—C14—C15—C16	−0.6 (4)
C1—C2—C7—O2	−179.2 (2)	C14—C15—C16—C17	0.4 (4)
C3—C2—C7—N1	−179.9 (2)	C15—C16—C17—C12	0.2 (4)
C1—C2—C7—N1	0.4 (3)	C13—C12—C17—C16	−0.5 (3)
N1—N2—C8—C9	178.94 (17)	C11—C12—C17—C16	178.0 (2)

### Hydrogen-bond geometry (Å, °)

**Table:**

*D*—H···*A*	*D*—H	H···*A*	*D*···*A*	*D*—H···*A*
O1—H1A···N1	0.82	1.86	2.586 (2)	147
O5—H5B···N5	0.82	1.79	2.519 (2)	148
C19—H19A···O2^i^	0.96	2.58	3.525 (3)	170
O3A—H···O5^ii^	.	.	2.643 (3)	.

Symmetry codes: (i) −*x*, −*y*+1, −*z*+1; (ii) *x*+1/2, −*y*+1/2, *z*+1/2.
